# Achieving Triply Periodic Minimal Surface Thin-Walled Structures by Micro Laser Powder Bed Fusion Process

**DOI:** 10.3390/mi12060705

**Published:** 2021-06-16

**Authors:** Shuo Qu, Junhao Ding, Xu Song

**Affiliations:** Department of Mechanical and Automation Engineering, The Chinese University of Hong Kong, Shatin, Hong Kong, China; qushuo@link.cuhk.edu.hk (S.Q.); jhding@link.cuhk.edu.hk (J.D.)

**Keywords:** thin-walled structure (TWS), triply periodic minimal surface (TPMS), laser powder bed fusion (LPBF), process parameter window, heat dissipation capability

## Abstract

Recently, triply periodic minimal surface (TPMS) lattice structures have been increasingly employed in many applications, such as lightweighting and heat transfer, and they are enabled by the maturation of additive manufacturing technology, i.e., laser powder bed fusion (LPBF). When the shell-based TPMS structure’s thickness decreases, higher porosity and a larger surface-to-volume ratio can be achieved, which results in an improvement in the properties of the lattice structures. Micro LPBF, which combines finer laser beam, smaller powder, and thinner powder layer, is employed in this work to fabricate the thin-walled structures (TWS) of TPMS lattice by stainless steel 316 L (SS316L). Utilizing this system, the optimal parameters for printing TPMS-TWS are explored in terms of densification, smoothness, limitation of thickness, and dimensional accuracy. Cube samples with 99.7% relative density and a roughness value of 2.1 μm are printed by using the energy density of 100 J/mm^3^. Moreover, a thin (100 μm thickness) wall structure can be fabricated through optimizing parameters. Finally, the TWS samples with various TPMS structures are manufactured to compare their heat dissipation capability. As a result, TWS sample of TPMS lattice exhibits a larger temperature gradient in the vertical direction compared to the benchmark sample. The steady-state temperature of the sample base presents a 7 K decrease via introducing TWS.

## 1. Introduction

Cellular structures have shown attractive multifunctionalities due to the complicated geometries and scale effect [1]. As a group of mathematically defined cellular structures, the triply periodic minimal surface (TPMS) lattice structure has fascinating properties which possess smooth surfaces without sharp corners or joints and a large surface area to volume ratio. It has been demonstrated that TPMS architecture holds great potential in revolutionizing many engineering applications such as tissue engineering, catalytic substrates, and heat exchangers [2]. However, the fine features and sophisticated surfaces of TPMS pose a great challenge to conventional manufacturing techniques.

The emergence of additive manufacturing (AM), or commonly known as 3D printing, is proven to be a feasible approach to fabricate complex models like TPMS [3], which accelerates the research on thin-walled TPMS applications. The decrease in the surface thickness implies an increase in porosity which leads to a biocompatible, biodegradable tissue structure with both surface and structural compatibility [4]. Furthermore, the TPMS structure with higher porosity, or equivalently lower relative density, gives bone scaffolds higher permeability, which promotes bone repair [5]. In terms of convective heat transfer, the heat dissipation of TPMS structure is typically proportional to the surface area, meaning that thinner walls are more beneficial under convection [6]. As to the heat exchangers applications, the TPMS structure necessitates a leak-free design, and thin walls minimize resistive-thermal losses [7]. Moreover, gradient thickness [8] and cell size [9] have been attempted to study the permeability and elastic properties, which demand a low value of thin-wall thickness that can be printed as the desirable design reference. Therefore, the thin-walled structure (TWS) of TPMS design is a promising structure to be investigated.

Plastic TPMS lattice structures have been attempted by material extrusion [10] and material jetting [11]. However, the small printing scale and low resolution restrict their further applications. At present, metallic TPMS structure printing is extensively employed thanks to its excellent repeatability and stability [12,13]. Compared with other metallic AM techniques, laser powder bed fusion (LPBF) is well-suited for fabricating the TPMS structures with high resolution and good dimensional accuracy [2]. Generally, most LPBF machines hold a typical accuracy of ±50 μm [14] and a minimum feature size of 200 μm [15]. The printed components with TWS feature design always show an increase in the relative density as the adjacent powder melts and sticks to the surface [16]. This phenomenon becomes obvious when the wall thickness thins down to the limit of the machine. The reason is that the interaction between solidified structure and the powder bed increases as the surface area to volume ratio increases, where this interaction usually leads to the partial melting of the loose powder during the solidification of the printed layers [17]. In addition, the complete and smooth TWS is hard to be manufactured for the TPMS structure, which has plenty of curved surfaces and overhangs due to thermal stress from the material during fabrication [18]. It is demonstrated that 10% relative density of Diamond(D-) type of skeletal TPMS structure using titanium alloy Ti6Al4V powder failed to build due to a 280 μm strut diameter, which is too fragile to resist the distortion of residual stress [19]. Therefore, the successful fabrication of TPMS TWS requires a stable material with a wide process window to achieve the complicated lattice. Recently, stainless steel 316 L (SS316L) has become one of the most common choices in process parameters research [20,21,22] and exhibits lower residual stresses than Ti-6Al-4V [23]. Furthermore, SS316L owns good corrosion resistance, high strength, and process stability [24], which can promote considerable development in TWS applications. In addition to the choice of materials, the TWSs fabrication is also related to process parameters and LPBF system configuration. It is because that the feature size mainly depends on the size of the molten pool during processing [17], resulting from the stair-step effect [25]. The molten pool size is attributed to the process parameters [26] and LPBF system properties consisting of powder size, layer thickness and laser beam size. The diameter of the spherical powder used by the LPBF system is commonly in the range of 10–60 μm [27], and the layer of 20–60 μm thickness is frequently chosen. Recently, the micro LPBF has been proposed and reported [28,29,30], which possesses finer microstructure [28,29,30] and smaller distortion [28,29,31] than conventional ones. Therefore, a micro LPBF system based on Han’s laser machine frame M100 with 25 μm laser beam, 10 μm slice layer and 5–25 μm powder is employed to achieve a finer structure in this work, which is beneficial to the fabrication of TWS.

In our work, the optimal process parameters of LPBF for TWS TPMS are determined by printing cube samples and thin-walled cylinders. The density and roughness of cubes are evaluated by using different parameters set. Then we focus on the optimization of parameters that lead to a dense and smooth TWS through varying the laser power, scanning speed, and hatch distance (HD). The G-type shell-based TPMS TWS samples with variable cell size and thickness are fabricated to verify the feasibility of optimal parameters. Finally, the heat dissipation capability of TPMS TWS samples is demonstrated in the form of pseudo heatsinks.

## 2. Materials and Methods

### 2.1. Geometric Modeling of TPMS Structures

**Figure 1:**
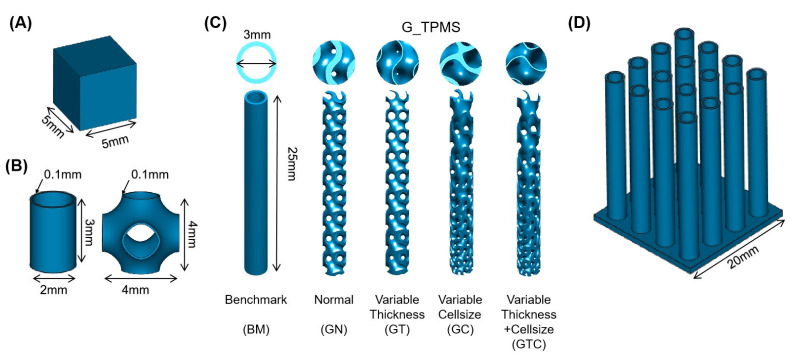
Design models: (**A**) 5 × 5 × 5 mm^3^ cube for energy density parameter optimization; (**B**) 0.1 mm wall-thickness cylinder and 0.1 mm wall-thickness of P-type TPMS samples for parameters optimization of TWS; (**C**) Different sample design with BM/GN/GT/GC/GTC structures; (**D**) Pseudoheatsink example design.

Figure 1A,B show the models generated by Materialise Magics^®^ (Version 24.1, Materialise NV, Leuven, Belgium) and Matlab software (Version R2020a, MathWorks Inc., Natick, MA, USA), which were used in the parameter optimization process. The benchmark (BM) sample was composed of 16 cylinders (see Figure 1C), which were placed in order of 4 by 4 on a 20 mm × 20 mm plate with a thickness of 1 mm (see Figure 1D). Then, Matlab was used to generate the G-type TPMS sheet network structures with a normal type (GN), thickness variable type (GT), cell variable type (GC), cell size and thickness variable type (GTC). The G sheet lattice was generated by enclosing two surfaces, which are defined by the following function:

(1) $$\phi_{G}\left(x,y,z\right)=\sin\left(wx\right)\cos\left(wy\right)+\sin\left(wz\right)\cos\left(wx\right)+\sin\left(wy\right)\cos\left(wz\right)=C\left(x,y,z\right)$$

where $w=\frac{2\pi }{L\left(x,y,z\right)}$, $L\left(x,y,z\right)$ denotes the length of a unit cell and $C\left(x,y,z\right)$ controls the interface surface. Variable cell size and thickness were constructed by adjusting the $L\left(x,y,z\right)$ and $C\left(x,y,z\right)$.

The model parameters of samples were indicated in Table 1. The specimens in this work were assembled, sliced and generated as job files in the Materialise Magics software for printing. The model parameters of samples are indicated in Table 1. The relative density of each model was set as 40% and the thinnest thickness of the GT/GTC model was given as 0.1 mm.

### 2.2. Materials and Processing

All specimens in this work were fabricated by a self-developed micro LPBF machine, Han’s Laser M100µ (which is adapted from a Han’s Laser M100 platform), employing a Yb laser source (λ = 1.07 μm) with an improved 25 μm beam size and an upward powder feeding system with a rubber doctor blade. The fine powders are heated at 120 °C in a low vacuum environment (0.8 bar) for 2 h before recoating to prevent fine powder agglomeration. Furthermore, the powder feeding cylinder and printing cylinder have a positioning accuracy of 5 μm to achieve the thin layer thickness. SS316L powders (D50 = 16.27 μm) with 5–25 μm diameter sizes produced by gas atomization and 10 μm layer thickness were employed in the micro-LPBF system. Nitrogen protective gas was adopted due to the stability of SS316L, and the printing process was conducted when the oxygen concentration was under 500 ppm. We chose a 115 × 115 mm^2^ base plate fabricated by SS316L for enhancing the adhesion and minimize the thermal and metallurgical mismatch between specimens and plate. Density, roughness, and wall thickness were used to evaluate the process parameters to find the optimal parameters to build TWS of TPMS. Firstly, the parameters shown in Table 2 were employed to fabricate the cubes with the dimension of 5 mm × 5 mm × 5 mm. The energy density is calculated as follows:

(2) $$E_{D}=\frac{P}{v\times h\times t}\;$$

where $E_{D}$ is energy density, P is laser power, v is scanning speed, h is HD, t is layer thickness, which is 10 μm. $E_{D}$ is used commonly to guide parameter optimization in LPBF [32].

After determining the optimal energy density by characterizing the cube samples, a thinned wall cylinder with 0.1 mm thickness and a P-TPMS unit cell with a cell size of 4 mm and 0.1 mm thickness were chosen as the TWS models for dimension accuracy parameters optimization. The TWS cylinders and P-TPMS cells were fabricated by the parameter set of B1 shown in Table 3. The parameters of B1 had the same $E_{D}$ determined by the optimal result of A1–A3. In this work, no contour strategy was performed, and the hatch angle was 67°.

### 2.3. Characterization Methods

After printing, the specimens were cut from the baseplate by wire electrical discharge machining. Then they were cleaned by an ultrasonic cleaner immersed in ethanol of 98% concentration to remove the adhered loose powders. The density of specimens was obtained by the Archimedes method. Three cube samples of each parameter shown in Table 3 were measured for verifying the repeatability. Then, the roughness and morphology of the cross-section surface of polished specimens were characterized by RH-2000 High-Resolution 3D Optical Microscope, HIROX. The average roughness was measured from three different positions on each cube surface. The thickness of cylinder specimens is measured through optical microscopes, Leica DMI6000B. The heat sink performance was evaluated by a self-built testbed which consisted of a ceramic heating sheet of 30 × 30 × 2 mm^3^ with 12 W power with a maximum temperature of 200 °C. The TPMS and benchmark samples were put on the upper surface of the heating sheet, and two thermocouples were stuck under the testing samples to obtain the heat sink base temperature. To prevent the gap thermal resistance and enhance the heat conductance, there is a silicone pad between the sample and the heating sheet. A 25 × 25 mm^2^ fan blowing up with 3000 r/min was placed 45 mm above the heating sheet. A FLIR A655sc infrared camera was employed to observe the temperature distribution of samples.

## 3. Results and Discussion

### 3.1. Cube Quality

#### 3.1.1. Relative Density

**Figure 2:**
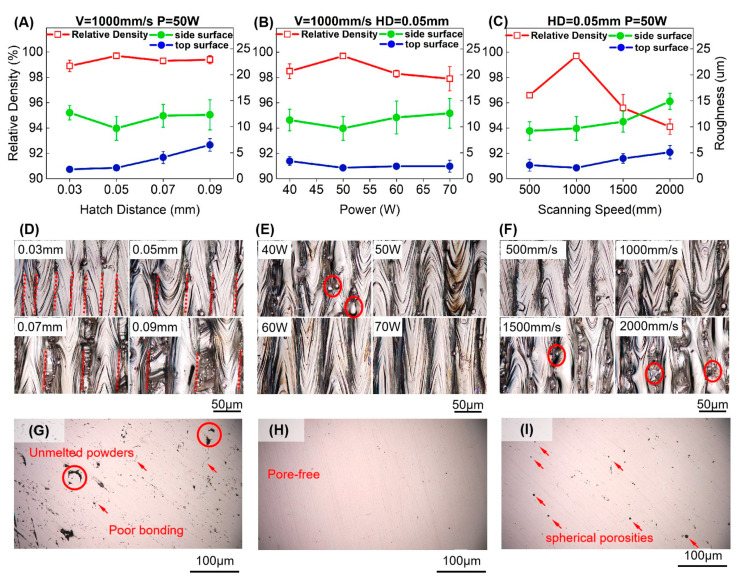
(**A**–**C**) The relative density and roughness of cubes with different energy density parameters; (**D**–**F**) Top surface morphology of cubes; (**G**–**I**) Polished side surface morphology of three process conditions: lack of fusion, dense, overheating.

Figure 2 shows the results of relative density, roughness, and the OM images of cubes printed by the corresponding parameters. The scanning direction and distance of laser tracks can be clearly observed on the cube top surfaces in Figure 2D–F. It can be summarized that the cubes printed with 50 W laser power, 1000 mm/s scanning speed, and 0.05 mm HD have the highest relative density of 99.7%.

Firstly, varying HD from 0.03 mm to 0.09 mm, the relative density shows a high value of more than 99% and changes slightly, as shown in Figure 2A. Although the increase in the track gap results in the discontinuity between tracks in the current layer, the remelting layer by layer during the process can densify the unmelted area of the previous layer due to the scan strategy of rotating 67°. Moreover, the 10 μm layer thickness employed in our work can enhance the remelting effect between layers to obtain a high relative density in various HDs.

Then, the cubes exhibit an increase and then a decrease in the relative density through adjusting laser power from 40 W to 70 W and scanning speed from 500 mm/s to 2000 mm/s in Figure 2B,C. The low relative density can be attributed to the insufficient melting for 40 W power and more than 1000 mm/s scanning speed. We can find the width of tracks will narrow down when speed increase, which also leads to a shallow molten pool [33]. As a result, the shallow molten pool remelts of fewer previously printed layers to leave plenty of powders and cracks in the cubes. Nevertheless, the relative density of cubes used by the power of more than 50 W or the speed of 500 mm/s shows a decrease because of the keyhole mode caused by high energy density, which leads to a collapse of the molten pool [34]. Figure 2G–I show the side surface of cubes polished in three process conditions: lack of fusion, dense and overheating. The lack of fusion is contributed by the low energy density calculated by Equation (2), and the overheating microstructure is caused by the high energy density. There are unmelted powders and poor bonding areas indicated in Figure 2G. Due to the inner powders filled in the pores, the low relative density caused by lack of fusion was overestimated slightly by Archimedes measurements. Moreover, the spherical porosities can be found in the overheating processing microstructure due to high energy input.

#### 3.1.2. Surface Roughness

As shown in Figure 2A, the roughness of the top surface increases gradually with the increasing HD, and the HD of 0.05 mm contributes to the lowest side surface roughness. In Figure 2D, the laser tracks with different degrees of overlapping can be observed on the top surface with different HDs. It is well known that surface roughness is determined by the conditions of both molten pools and unmelted powders. It is readily understood that the single molten pool is a convex surface instead of a plane due to surface tension. Therefore, the 0.03 mm HD attributes the lowest roughness due to the sufficiently overlapping tracks. We can easily observe the previous layer tracks between the gap of top surface tracks in Figure 2D, which explains that the increase in the roughness of surfaces printed by 0.07 mm and 0.09 mm HD.

In Figure 2B, the surfaces of specimens fabricated by parameters with different powers represent a low level of top surface roughness except for the parameter with 40 W. Moreover, the unmelted powder could be found on the surface printed by 40 W shown in Figure 2E. Then, it can be seen that surface roughness goes up when the scanning speed is more than 1000 mm/s in Figure 2C. When laser power is not high enough to melt all the surrounding powders or the scanning speed is too fast to form a stable molten pool, the laser track will generate splashing powders and leave spherical powders on the track surface. This phenomenon can be obviously seen in Figure 2E,F, in which the power is lower than 50 W and the scanning speed is larger than 1000 mm/s as labeled by circles in red. As a result, the roughness of the surface can increase with the increasing HD, decreasing power, or increasing scanning speed.

### 3.2. Simple Thin-Walled Structures

#### 3.2.1. Thickness Limit of TWSs

**Figure 3:**
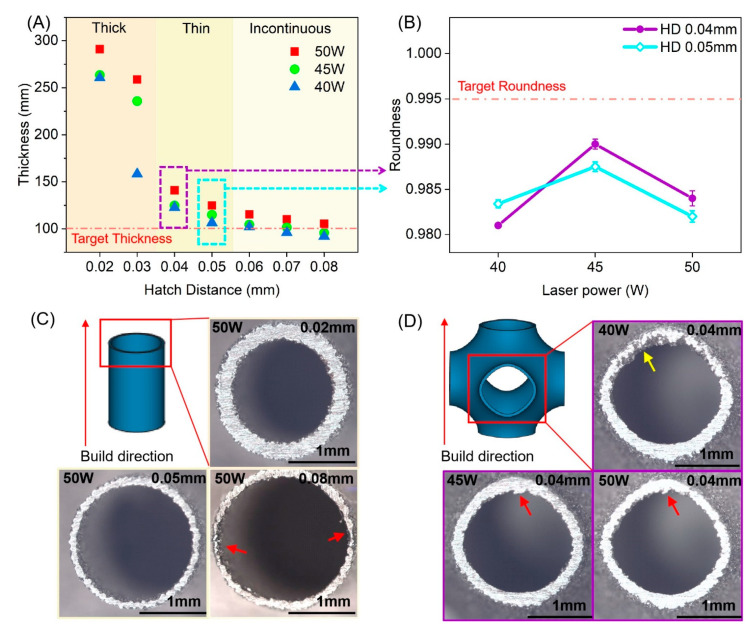
(**A**,**C**) The wall thickness and OM images of cylinders printed by 40–50 W power and 0.02–0.08 mm HD; (**B**,**D**) The roundness and OM images of side cross-section of P-TPMS TWS printed by 40–50 W power and 0.04/0.05 mm HD.

The wall thickness limit due to parameter variation is explored by examining the top cross-sections of the cylinder samples. Figure 3A shows that the thickness of cylinder specimens printed by the parameters set with 100 J/mm^3^ of the energy density of which HD varies from 0.02 mm to 0.08 mm and laser power varies from 40 W to 50 W, meaning the scanning speed is varied with the other two parameters to ensure the same energy density. When HD is 0.02/0.03 mm, the thickness of the cylinder is much larger than the target thickness. It is attributed to the head protrusion and tail depression of the melt track contributing greatly to the formation quality [35]. On the other hand, when the low HD is used, the laser spot will rescan the solidified track because the molten laser tracks are usually bigger than the spot size due to the heat diffusion. It is remarkable for fine beam in the low HD parameters of TWS, which leads to a ~0.3 mm thickness wall with a ~0.2 mm deviation from the model.

On the other hand, the wall thickness drops to about 100 μm as HD goes up to 0.08 mm. The cross-section morphology of the cylinder can be found in Figure 3C. As observed, the wall appears to be discontinuous when HD is 0.06 mm and above. It is noted that there is an inward offset from the design contour in the hatch area of the laser track as spot compensation. In this work, the spot compensation is set as 0.04 mm, meaning that the contour region on both sides of the wall is 0.04 mm in width. For the 0.1 mm wall-thickness cylinder, there is a hatch region of 0.02 mm width to fill with laser paths. It results in the disappearance of the laser path on some positions in the sliced file when the HD is much more than the width of the hatch region. This lack causes a discontinuous wall structure, as indicated by the red arrow in Figure 3C, which can be found in the specimens printed by HD of 0.06–0.08 mm. However, specimens printed by HD lower than 0.05 mm have no discontinuous wall. It is attributed that the remelting for the previous layer and the hatch angle of 67°, which ensures the coverage of the previously unmelted region path. As a result, at the energy density of 100 J/mm^3^, HD of 0.04/0.05 mm is the optimal parameter for thin wall thickness.

#### 3.2.2. Dimensional Accuracy of TWSs

After obtaining the process limitation of thickness, we put the focus on the dimension accuracy of TWS. Thermal stress, which results from the rapid cooling rate [36], is responsible for the distortion of the structure. Note that this deviation deteriorates on the TWS with overhangs such as a circle or low angle wall, which is known as the staircase effect [25]. Thus, the roundness of the side cross-section is an appropriate parameter to illustrate the dimension accuracy on TWS.

Figure 3B shows the roundness of the side structure of as-printed P-TPMS TWSs with parameters of 40–50 W power and HD 0.04/0.05 mm obtained from the above chapter. The roundness of side structure [37] is used to quantitatively characterize dimension accuracy of TWSs, which is defined by:

(3) $$Roundness=\frac{4\pi A}{P^{2}}$$

where A is the area of the side cross-section, and P is the perimeter of the area. As defined, the closer the roundness value is to 1, the rounder the shape is. Because the side cross-section of P-TPMS TWS is not a perfect circle, the model roundness is 0.995.

In Figure 3B, it is found that samples printed by 0.04 mm of HD have better roundness with 45/50 W power parameters than 0.05 mm of HD. The narrower HD accompanied by higher scanning speed causes a shallower pool resulting in lower-dimensional deviation [38]. On the other hand, it suggests that the power of 50 W can lead to a more serious deformation on low inclination surfaces than the power of 45 W from Figure 3B. Though the parameter combinations are of the same energy density, the higher power can still generate the deeper molten pool [39], the shrinkage of which causes more residual stress.

In Figure 3D, the OM images of side structure printed by 0.04 mm HD and three different powers of 40/45/50 W are shown. The red arrow points to the deformation of the overhang structure. It is found that all the side structures have some distortion compared to models. The yellow arrow indicates pores and cracks in the surface, which are observed in specimens by 40 W power. The side cross-section morphology of 40 W is caused by insufficient melting. It can be attributed to the lower inclination wall resulting in irradiation of laser on the more powder-supported zone rather than the solid-supported zone, which forms a larger molten pool [38] and leads to a lower surface quality [40].

### 3.3. TPMS Thin-Walled Structures

**Figure 4:**
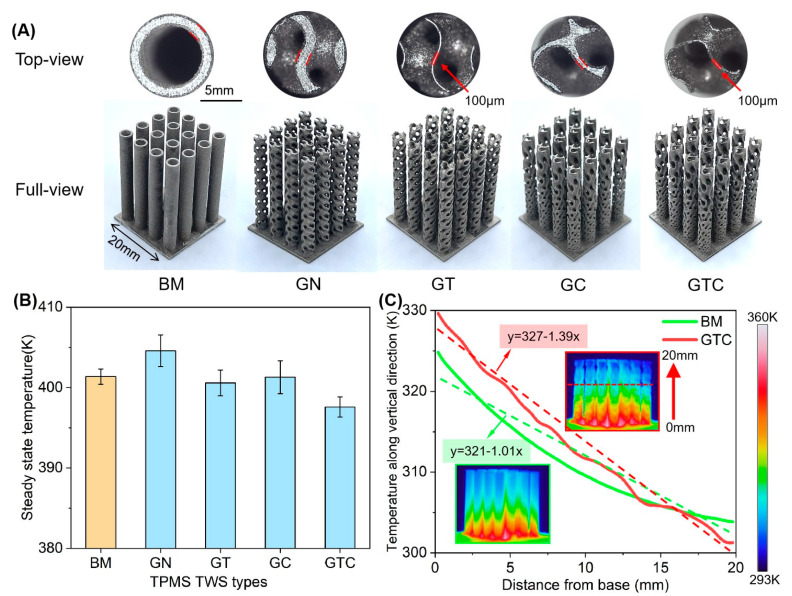
(**A**) As-printed samples of BM and TPMS structures and thickness comparison; (**B**) The steady-state temperature of base cooled by different samples; (**C**) The average temperature curves of cross-section on the vertical direction from heater; temperature distribution measured by an infrared camera as shown in the thermal image inserts.

Figure 4 exhibits the result of the fabricated TPMS TWSs and the heat dissipation experiment. In the full view, five types of samples are shown, which are built by the optimal parameters mentioned in the previous section. The top surface can be observed from the top-view figures, in which the wall thickness is measured directly. The thickness of 100 μm is realized in the GT and GTC TWS. In Figure 4B, the base temperature of different samples is plotted to represent the heat dissipation capability of samples.

BM sample shows a steady-state temperature of 401.4 K, while the GTC sample, which is obtained by adjusting cell thickness and cell size, exhibits the best heat dissipation capability and achieves a base temperature of 397.6 K. Furthermore, the results of GT and GC show a marginally lower than BM, respectively, 400.6 K and 401.3 K. Then, the temperature of GN, namely 404.6 K, is 7 K higher than that of GTC, which indicates that the participation of G-TPMS TWS improves the heat dissipation capability.

In Figure 4C, the temperature variation along the vertical direction of BM and GTC samples is plotted, and the distribution of heat is shown by the infrared thermal imaging camera. It is observed that the GTC has a higher temperature (329.6 K) in the base and a lower temperature (301.3 K) on the top compared with the base (324.8 K) and the top (303.9 K) of the BM sample. From the thermal image inserts, we can find that more heat is gathered at the base of the GTC sample than the BM sample, which is attributed to the larger cell thickness and smaller size at the bottom to improve the heat conduction from the heater to samples. The results of linear fitting of temperature curves indicated by dashed lines are shown, in which the slopes exhibit the temperature gradient along the vertical direction of TWSs.

In addition, the top of GTC sample shows a lower temperature due to that TWS promotes the convection heat dissipation [6]. The temperature difference between top and base of GTC sample is 28.3 K, 7.4 K higher than BM sample. Overall, TWS of TPMS shows an improved performance because of thin wall thickness reduction.

## 4. Conclusions

In this work, the optimal parameters of TWS fabricated by SS316L with micro LPBF are explored by cubes and thin-walled structures through adjusting laser power, scanning speed, and HD. Relative density, surface quality, thin-wall process limitation, and dimensional accuracy of components are studied. Utilizing the optimal parameters, the TWS of TPMS samples are fabricated to compare the heat dissipation capability with the traditional design. The main conclusions are stated as follows:

(1) The energy density of 100 J/mm^3^ proves to be the optimal choice by comparing relative density and surface roughness. Relative density of 99.7% is achieved by applying parameters with laser power of 50 W, HD of 0.05 mm, scanning speed of 1000 mm/s.
(2) In the energy density of 100 J/mm^3^, HD of 0.04/0.05 mm contributes to a thin and continuous wall of 100 μm thickness. In this process window, the parameter with the laser of 45 W, HD of 0.04 mm, scanning of 1125 mm/s has the best dimension accuracy.
(3) The TPMS TWSs with variable cell thickness and size structure exhibit the most excellent performance in heat dissipation due to the fine structure promoting convection heat dissipation. The difference of base temperature cooled by GTC and GN is 7 K via introducing the TWS. The temperature gap between the top and bottom of the GTC sample is 28.3 K, which is 7.4 K higher than the BM sample, representing a better heat dissipation capability.

## Figures and Tables

**Table 1:** Model parameters of samples.

Type	Smallestthickness (mm)	Relative Density	Surface Area (mm^2^)
BM	0.34	40%	422.6
GN	0.43	40%	430
GT	0.10	40%	425.6
GC	0.27	40%	509
GTC	0.10	40%	502.2

**Table 2:** The parameters adopted for optimization of printing cubes with different energy densities.

Parameters	Laser Power (W)	Scanning Speed (mm/s)	Hatch Distance (mm)	Energy Density (J/mm^3^)
A1	50	1000	0.03/0.05/0.07/0.09	167/100/71/56
A2	40/60/70	1000	0.05	80/120/140
A3	50	500/1500/2000	0.05	200/67/50

**Table 3:** The parameters adopted for optimization of printing TWS with the same energy density.

Parameters	Laser Power (W)	Scanning Speed (mm/s)	Hatch Distance (mm)	Energy Density (J/mm^3^)
B1	40/45/50	varied by other parameters	0.02/0.03/0.04/0.05/0.06/0.07/0.08	100
